# Severe colic in a newborn dairy calf caused by a large colostrum curd: a case report

**DOI:** 10.3389/fvets.2024.1348084

**Published:** 2024-06-19

**Authors:** Donald C. Sockett, Andrea E. Pohly, Kathleen M. Deering, Ryan M. Breuer

**Affiliations:** ^1^Wisconsin Veterinary Diagnostic Laboratory, University of Wisconsin-Madison, Madison, WI, United States; ^2^Department of Pathobiological Sciences, School of Veterinary Medicine, University of Wisconsin-Madison, Madison, WI, United States; ^3^Department of Medical Sciences, School of Veterinary Medicine, University of Wisconsin-Madison, Madison, WI, United States

**Keywords:** abomasum, newborn, abdominal distension, postmortem evaluation, colostrum guidelines, esophageal feeder

## Abstract

A newborn female, Holstein calf weighing approximately 38.5 kg developed severe, persistent colic caused by a large colostrum curd located within the calf’s abomasum. Based upon 10% body weight, the calf had been fed 4 liters (L) of first-milking colostrum approximately 30 min after birth and an additional 2 L of first-milking colostrum 6 h after the first feeding. Both the first and second feedings used an esophageal tube feeder to deliver the colostrum. Colic developed shortly after the second colostrum feeding. The affected calf did not respond to on-farm supportive medical therapy and was humanely euthanized by a penetrating captive bolt approximately 22 h after the onset of colic. This on-farm colostrum feeding protocol is routinely observed in the current dairy industry. This case demonstrates calves that are fed large volumes of colostrum during a relatively short window of time may develop a large, firm colostrum curd within the abomasum that causes abdominal distension, colic, and occasional death. There is an urgent need for prospective analytical studies that determine the optimal immunoglobulin mass (g/L) and the ideal volume of colostrum fed to newborn calves for both the first and second colostrum feedings within the most beneficial time frame. Guidelines should be developed that minimize complications that adversely affect calf health and well-being while ensuring the successful transfer of passive immunity.

## 1 Introduction

A comprehensive colostrum program is the most important management factor that affects the health and well-being of calves (1–3). Newborn calves are born agammaglobulinemic and depend on the successful transfer of passive immunity for optimal health (3, 4). Current best practices recommend that dairy calves be fed 4 L (10–12% of birth weight) of high-quality colostrum (Brix score ≥ 22%) within 2 h of birth, followed by a second 2 L colostrum feeding 10–12 h after birth (1, 5, 6). This feeding protocol is commonly practiced in the dairy industry. Many calves will not voluntarily drink 4 L of colostrum for the first feeding, which necessitates the use of an esophageal tube feeder to deliver the colostrum to the newborn calf (1, 6–8). Feeding colostrum using an esophageal tube feeder is not risk-free. Complications involve aspiration pneumonia and resistance by the calf increases the likelihood of pharyngeal and esophageal trauma (7, 9, 10). Abdominal distension and colic may arise after the second colostrum esophageal tube feeding, which is one of the reasons many calf care specialists recommend that calves only be allowed to drink the second feeding. A survey of calf care providers and calf managers representing 53 different dairy operations conducted in November 2023 (personal communications, DS) found that less than 10% of female Holstein calves will voluntarily drink 2 L of colostrum 10–12 h after birth when they are initially esophageal tube fed 4 L of colostrum within 2 h of birth. With so many calves refusing to drink 2 L of colostrum for the second feeding, many calf care providers do not offer a second colostrum meal, or they deliver 2 L of colostrum using an esophageal tube feeder.

## 2 Patient information

A 10-h-old, female, Holstein calf weighing approximately 38.5 kg presented on-farm to an in-field herd veterinarian for severe colic following industry-recommended guidelines for colostrum delivery. Delivery and birth of this heifer calf were unremarkable. There was neither dystocia nor prolonged time to delivery, and the calf was born without calf care provider assistance. Prior to veterinary physical examination, the calf was fed 4 L of colostrum 30 min after birth, with a second 2 L feeding given 6 h after the first feeding. On-farm equipment that pasteurizes colostrum in either 4 L or 2 L disposable bags was used for feeding the affected calf. The colostrum was delivered using a single-use plastic esophageal tube feeder for both feedings (Perfect Udder®, Dairy Tech Inc., Windsor, Colorado, USA). The calf was in sternal recumbency for the first feeding and stood without assistance for the second feeding. During both feedings, the calf was bright, alert, and responsive.

## 3 Clinical findings

Upon on-farm physical examination, the laterally recumbent calf exhibited signs of immense pain and discomfort, including tachycardia (>150 beats per minute) and tachypnea (>75 breaths per minute). The normal reference ranges for newborn calf heart and respiratory rates are 80–120 beats/min and 24–36 breaths/min, respectively (11). The calf’s temperature was normothermic. The mucous membranes were pink and moist, and there was no scleral injection. Skin tent, although moderately conducive for hydration status assessment, did not indicate signs of dehydration. The calf had pronounced vocalization, grunting, and profound abdominal distention. The abdominal distension and colic signs developed 1–2 h after the esophageal tube feeder was used to deliver 2 L of colostrum at the second feeding.

## 4 Therapeutic intervention

Supportive medical treatment was provided by the herd veterinarian, on-farm after the initial physical examination, which included intravenous (IV) fluid therapy (4–5 L of lactated Ringer’s solution at a rate of ~100 mL/kg/h) and IV flunixin meglumine (2.2 mg/kg) administered once per the manufacturer’s recommendations. Euthanasia was elected due to the lack of response to medical intervention, the worsening condition creating calf health and welfare concerns, and the extreme toll the suffering calf placed upon the calf care providers. The calf was humanely euthanized using a penetrating captive bolt approximately 20 h after the initiation of medical treatment.

This was the sixth to eighth newborn calf in the fall of 2021 that displayed similar physical signs prior to death on this ~1,500 cow dairy farm shortly after the second colostrum administration. Furthermore, in 2021, the herd veterinarian had observed the same complications in 2–3 other dairy herds that followed the same colostrum feeding protocol.

## 5 Postmortem examination

**Figure 1:**
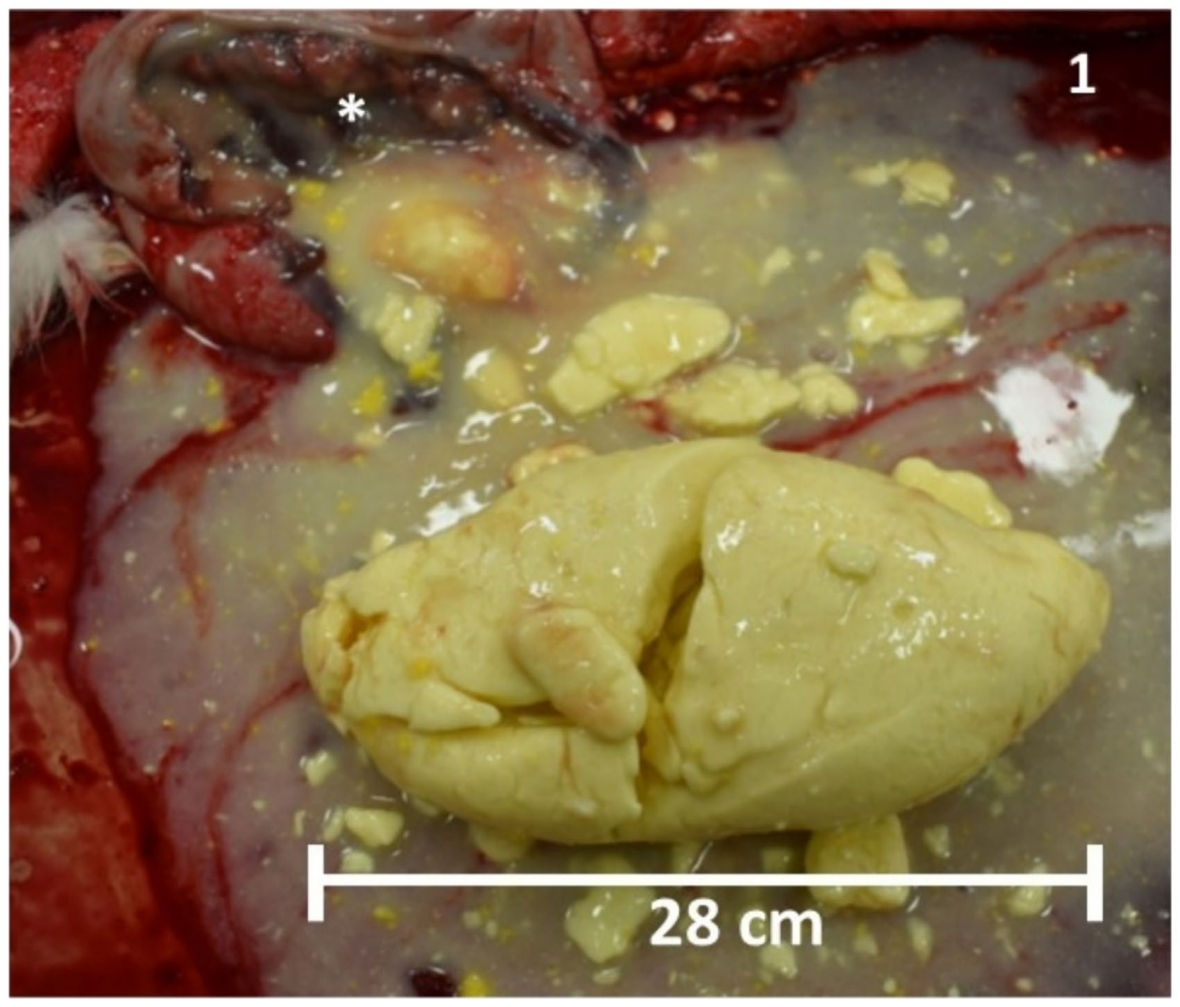
Postmortem abomasal colostrum clot. Photo of a very solid, yellow colostrum curd (28.0 cm in length, 15.4 cm in height) removed from the abomasum (white asterisk) of a 39.5 kg female, Holstein calf. Microscopic changes in the abomasum are limited to moderate vascular congestion.

One of the services provided to the livestock industry by veterinary diagnostic laboratories is postmortem examination and ancillary testing of recently deceased animals. In October 2021, the Wisconsin Veterinary Diagnostic Laboratory (WVDL) received the 30-h-old, female Holstein calf described in this report for postmortem examination. The calf arrived at the diagnostic lab within 2 h of euthanasia. The deceased calf weighed 39.5 kg. A routine gross examination of the calf’s body was performed by a board-certified anatomical veterinary pathologist. There was no evidence of aspiration or upper airway obstruction nor evidence of congenital lesions (i.e., atresia coli), meconium retention, or calving/esophageal tube feeding trauma. Upon examination of the gastrointestinal tract, a 28.0 cm in length, 15.4 cm in height, solid, yellow colostrum curd was found within the abomasum (Figure 1). The underlying abomasal mucosa was reddened. Standard tissue samples (brain, heart, lung, thymus, liver, spleen, kidney, adrenal glands, urinary bladder, urachus, synovial tissues, tongue, forestomach, abomasum, and small and large intestine) were collected in 10% neutral-buffered formalin for microscopic examination. Real-time PCR diagnostics were performed on both the lung and intestinal tissues to rule out common infectious agents that are implicated in bovine respiratory disease (bovine viral diarrhea virus, infectious bovine rhinotracheitis virus, bovine respiratory syncytial virus, bovine respiratory coronavirus, *Bibersteinia trehalosi*, *Histophilus somni*, *Mannheimia haemolytica*, *Pasteurella multocida*, and *Mycoplasmopsis bovis*) and neonatal calf diarrhea (*Cryptosporidium* spp., *Salmonella* spp., *E. coli* K99^+^, group A bovine rotavirus, and bovine coronavirus). All tissue samples examined microscopically were within normal limits, and PCR testing did not detect infectious agents. The pathologist concluded that the cause of the colic was the large colostrum curd located within the abomasum.

## 6 Discussion

To the authors’ knowledge, this is the first report that describes marked abdominal distension and unrelenting colic caused by an extremely large colostral curd formed within a calf’s abomasum. Calves, such as foals, are simple-stomached animals at birth (12, 13). The newborn calf’s reticulum, rumen, and omasum are underdeveloped and inactive for at least the first 2 weeks of its life (13). Concerns for abomasal health and function arise when the abomasum is over-distended with a large colostrum curd. Large curds usually develop after the second feeding, particularly when female, Holstein calves are tube-fed 2 L of colostrum less than 8 h after they were fed 4 L of colostrum. The formation of profuse colostrum curds occurs because large curds of colostrum are slowly digested over a time period of 12–18 h. When a calf is fed a second feeding of colostrum in a relatively short period of time, this excessively adds to the undigested curd that has already formed in the abomasum (13).

Some calf care providers, calf managers, and veterinarians have observed discomfort and abdominal distension in newborn female, Holstein calves shortly after the first feeding of 4 L of colostrum delivered by an esophageal tube feeder (personal communications, DS and RB). This observation is not surprising because when calves are not allowed to drink colostrum, the esophageal grove is not formed and the colostrum is deposited directly into the reticulorumen, which is half the size of the abomasum at birth (13). Chapman et al. reported that when 1–17-day-old Holstein calves are fed 2 L of a glucose and electrolyte solution delivered by an esophageal tube feeder, the rumen holds up to 400 mL of solution before starting to overflow into the abomasum (14, 15). Fortunately, the abdominal pain caused by feeding 4 L of colostrum to newborn calves is short-lived (≤30–60 min) because most of the colostrum is delivered from the forestomach to the abomasum and small intestine within 3 h (1, 16).

A study that established abomasal volumes, location, and emptying rates in calves by transabdominal ultrasonography showed that young, 7–30-day-old Holstein bull calves, when allowed to nurse a commercial milk replacer diet in 250–500 mL increments, had a mean measured abomasal dimensional length of 19.3 ± 4.1 cm and height of 12.4 ± 1.4 cm after ingesting 3 L (17). The abomasal size described in the publication is smaller than the size of the abomasal curd described in this report. Janssen et al., who reviewed the role of gastric motility in humans as a central component of hunger and satiety, reported that distention (by nutrient or non-nutrient meals, or by intragastric ballooning) of the human monogastric stomach induced a satiating effect that ranged from sensations of feeling full to feeling pain (18, 19). In addition, some calf care providers and veterinarians have observed that female, Holstein calves that are fed large volumes (≥4 L) of colostrum for the first meal, often will not nurse for 18–36 h post-colostrum feeding (20). The health effects and potential risks for newborn calves with an extended lapse in time between meals are unknown at this time, and further investigation is needed.

The calf care providers for this case followed on-farm protocols for colostrum feeding that are based on current best practice recommendations for the industry (1, 20). Previous publications have suggested that feeding a smaller volume of colostrum is preferable because there is a higher efficiency of absorption of bovine immunoglobulin (21–24). Studies have also shown that colostrum curd formation in the abomasum is beneficial to the absorption of immunoglobulins because it allows for the separation of the curd from the immunoglobulin-rich whey protein component of colostrum. The whey fraction, containing the immunoglobulins, is quickly released from the abomasum into the small intestine for absorption. Thus, the absence of colostrum curd formation can reduce the efficiency of immunoglobulin absorption (25, 26).

To form a colostral curd, prochymosin is converted to chymosin (rennet) under acidic (pH 4–5) conditions (25). Due to wide variations in calves’ curd forming ability and differences in apparent efficiency of absorption, recommendations to feed larger volumes (6–8 L) of colostrum in two meals to female, Holstein calves within the first 10–12 h of life (1, 6, 27) may present detrimental effects to the calf. In some calves, large-volume colostrum feeding causes the formation of a sizeable colostral curd within the abomasum that takes an extended period of time to digest, thus reducing abomasal space for subsequent feedings (23, 25). Feeding a smaller colostrum volume (2–3 L) that contains a higher concentration of immunoglobulin G (≥75 g/L), will increase the efficiency of immunoglobulin absorption and prevent the formation of extremely large colostrum curds like the one described in this case report. This smaller volume may improve forestomach and abomasal health and shorten the period of time that calves will not drink a liquid meal post-initial-colostrum feeding (20).

Severe colic, as described in this report, occurs infrequently and many calf care providers and practicing veterinarians accept this as an unfortunate complication of ensuring dairy calves receive an adequate mass of immunoglobulin for successful transfer of passive immunity (20). In Europe, force-feeding colostrum using an esophageal tube feeder can be controversial and, in some countries, tube feeding is prohibited except for medical reasons (28). Abdominal distension and colic caused by large-volume colostrum feeding can be mitigated by gaining further knowledge of how to appropriately assess colostrum quality (20). Additionally, calf care providers can be trained on the appropriate administration of colostrum, which includes identification of the risks and complications of overfeeding with an esophageal tube feeder (7–9, 20).

It is the authors’ robust opinion that feeding a smaller volume of colostrum that contains a sufficient mass of immunoglobulin to have the successful transfer of passive immunity is preferable to current colostrum feeding guidelines (1, 3, 6). Further research and investigation are needed to determine optimal immunoglobulin mass relative to each calf’s birth weight, the volume of colostrum fed to newborn calves for the first and second colostrum meals, and the timing of each feeding. Once new guidelines are established, social media platforms, industry publications, and scientific articles are excellent means of reaching a wide stakeholder audience for delivering information on colostrum feeding, animal husbandry, and management practices (29).

## 7 Conclusion

The practical consequences of feeding dairy calves large volumes of colostrum during a relatively short time frame, as presented in this case report, illustrate the need for investigation into colostrum feeding practices for newborn dairy calves. Feeding smaller volumes of high-quality first-milking colostrum (immunoglobulin G concentration ≥ 75 g/L), or extending the time interval between the first and second colostrum feedings to not less than 8–10 h may reduce the incidence of excessive abomasal distension causing discomfort and colic in newborn calves. Investigations that focus on different methods of colostrum delivery and which determine the ideal mass of immunoglobulin, length of time between the first and second colostrum feeding, and the volume of colostrum that should be fed to newborn calves based on the calf’s birth weight, are urgently needed. New guidelines must minimize colostrum feeding complications that adversely affect calf health and well-being, while still ensuring the successful transfer of passive immunity.

## Data availability statement

The original contributions presented in the study are included in the article/supplementary material, further inquiries can be directed to the corresponding author.

## Ethics statement

Ethical review and approval was not required for the study involving animals in accordance with the local legislation and institutional requirements. Written informed consent from the owners was not required to participate in this study in accordance with the national legislation and the institutional requirements. Written informed consent was obtained from the owners of the animals for the publication of this case report.

## Author contributions

DS: Conceptualization, Data curation, Investigation, Project administration, Supervision, Writing – original draft, Writing – review & editing. AP: Data curation, Investigation, Visualization, Writing – original draft, Writing – review & editing. KD: Data curation, Investigation, Visualization, Writing – original draft, Writing – review & editing, Methodology, Supervision. RB: Data curation, Investigation, Project administration, Supervision, Writing – original draft, Writing – review & editing.
